# Mass balance and metabolite profiling of ^14^C-Zalunfiban in humans following single-dose subcutaneous administration

**DOI:** 10.1080/00498254.2025.2597225

**Published:** 2025-12-15

**Authors:** Robert B. MacArthur, Sem A. O. F. Rikken, Spandana R. Vootukuri, Barry S. Coller, C. Michael Gibson, Christopher B. Granger, G. Montalescot, Jurriën M. ten Berg, Murray P. Ducharme, Arnoud W. J. van ‘t Hof

**Affiliations:** a Department of Pharmacy Services, Rockefeller University Hospital, New York, NY, USA;; b Department of Cardiology, St. Antonius Hospital, Nieuwegein, The Netherlands;; c Cardiovascular Research Institute Maastricht (CARIM), Maastricht, The Netherlands;; d Laboratory of Blood and Vascular Biology, Rockefeller University, New York City, NY, USA;; e Boston Clinical Research Institute, Boston, MA, USA;; f Department of Cardiology, Duke University School of Medicine, Durham, NC, USA;; g Sorbonne Université, ACTION Study Group, ICAN-Institut de Cardiologie, Hôpital Pitié-Salpêtrière (AP-HP), Paris, France;; h Department of Cardiology, University Medical Center Maastricht, Maastricht, The Netherlands;; i Learn and Confirm Inc, Montréal, Canada

**Keywords:** Drug absorption, drug excretion, drug metabolism, 14C-radiolabeled, GPIIb/IIIa inhibitor, mass balance, pharmacokinetic, zalunfiban, STEMI

## Abstract

1. The human metabolic and excretion profile of zalunfiban, a novel glycoprotein IIb/IIIa inhibitor, was studied in a phase 1 clinical trial.^14^C-zalunfiban was administered subcutaneously as a single fixed dose (9.5 mg with 5 μCi total radioactivity).
2. Zalunfiban whole blood concentrations were measured using liquid chromatography–mass spectrometry. ^14^C-zalunfiban and metabolites were measured using liquid scintillation counting and accelerator mass spectrometry in whole blood, urine, and faeces. Eight participants were enrolled. Zalunfiban was well-tolerated.
3. Following injection zalunfiban was detectable within 5 minutes and the last measurable concentration was observed within 4 hours. The median T_max_ was 0.25 hours and mean half-life was 0.96 hours. Zalunfiban accounted for 35.6% of total whole blood radioactivity AUC at 4 hours. The single major identified metabolite was des-gly-zalunfiban (M1), which has <1% of zalunfiban’s antiplatelet activity. M1 was the primary urinary metabolite (52.98% of dose) with minor amounts of zalunfiban (1.31%) detected. M1 was also the major metabolite in faeces (2.87%). Total dose recovery reached >90% by 240 hours.
4. Zalunfiban is rapidly metabolised to the nearly inactive M1 metabolite, which is excreted primarily in urine. Renal impairment, therefore, is unlikely to significantly prolong zalunfiban effects and dose adjustment in patients with reduced renal function may not be required.

## Introduction

Zalunfiban (2-amino-N-{5-[5-oxo-7-(piperazin-1-yl)-5H-[1,3,4]thia-diazolo[3,2-a]pyrimidin-2-yl]pyridin-3-yl}acetamide) is an investigational second-generation small-molecule platelet GP IIb–IIIa (integrin αIIbβ3) receptor inhibitor that is currently in a Phase 3 trial for first-point-of-contact treatment of ST-segment elevation myocardial infarction (STEMI) (NIH 2025; Rikken et al. 2023a). Zalunfiban inhibits fibrinogen binding, platelet aggregation, and platelet thrombus formation (De Luca et al. 2024). Early clinical trials have shown that following subcutaneous (SC) injection, zalunfiban has a rapid onset of action and a short half-life in whole blood (Bor 2021). It differs from the currently marketed intravenous GP IIb-IIIa inhibitors, tirofiban and eptifibatide, in its route of administration and in locking the receptor in its inactive conformation (Li et al. 2014; Rikken et al. 2023b). However, more information about the human metabolic and excretory pathways of zalunfiban is needed to better understand its pharmacokinetics (PK), pharmacodynamics (PD), and safety profile.

A human radiolabeled mass balance study is the single most direct method to obtain quantitative and comprehensive information about the absorption, distribution, metabolism, and excretion (ADME) of a drug. It can also help define the overall pathways of drug metabolism and excretion, as well as identify and quantify circulating metabolites. It can also provide information on which metabolites should be structurally characterised, and whether renal or hepatic studies or drug interaction studies should be considered (FDA Guidance Mass Balance Studies 2024a). The key safety, regulatory, and clinical pharmacology findings that can be derived from a well designed mass balance study are provided below (Table 1). Thus, the information that can be obtained is extremely valuable and therefore both the FDA and EMA recommend performing mass balance studies (Coppola et al. 2019).

To obtain this information we performed a mass balance study with a primary objective of assessing the cumulative excretion of ^14^C-zalunfiban in urine and faeces and characterising the PK in whole blood. Secondary objectives included identifying zalunfiban metabolites in whole blood, urine, and faeces, testing any primary whole blood metabolites for inhibition of platelet aggregation, and further assessing safety and tolerability in normal healthy volunteers.

## Materials and methods

### Study design

This was a Phase 1, single-centre, open-label, non-controlled, mass balance and metabolite profiling study of ^14^C-radiolabeled zalunfiban in health male volunteers (NCT04284995). The planned participant enrolment size was 8, with 2 additional alternate participants available on the morning of dosing. Following a 28-day screening period, all eligible participants were admitted to the research unit the evening before dosing. After fasting overnight, participants completed a standard breakfast prior to dosing on study day 1. After dosing, subjects fasted for 4 h. Fluids were permitted ad libitum starting 1-hour after dosing. The investigational product (IP) was injected SC on study day 1 and participants were housed for 11 days, which is past the final 240-hour sampling time, and then discharged following final evaluation. The clinical study was conducted at the Pharmaron Clinical Pharmacology Centre (Baltimore, MD) and the study protocol, consent forms and regulatory documents were reviewed and approved by the Advarra Institutional Review Board (Columbia, MD).

### Dose, preparation, and administration

All participants received the same fixed mass dose of SC 9.5 mg ^14^C-zalunfiban (5 μCi), in 0.5 mL of formulation solution. This zalunfiban dose was chosen to both ensure participant safety and to make certain the study results were clinically relevant. For the latter, by strict inclusion of participants with a body weight within the range of 73–99 kg, the zalunfiban doses were no higher than the highest administered in the currently ongoing Phase 3 study, which includes three groups of 0.110 mg/kg zalunfiban, 0.130 mg/kg zalunfiban, and placebo (NIH 2025). The ^14^C radiolabel dose of 5 μCi presented minimal radiation risk to healthy subjects while providing a high likelihood of meeting the study objectives.

Zalunfiban sterile solution in vials was provided by CeleCor Therapeutics, Inc (Del Mar, CA). ^14^C-zalunfiban was manufactured by Pharmaron, UK. The finished investigational product was compounded by the Pharmaron Research Pharmacy into individual doses and dispensed in 1 mL single-use syringes with a 25 G 5/8-inch needle.

Suitable SC injection sites included the upper arm or abdomen, in a location that did not interfere with study procedures. The injection site was required to be free of skin lesions, scars, tattoos, and bony prominences and had to be readily visible for monitoring. The injection was administered at a 45 to 90-degree angle to a pinched region of the skin. Following injection, a bandage was placed over the injection site. The bandage was later collected and tested for IP leakage by measuring bandage radioactivity.

### Population

Enrolled participants were required to meet all protocol inclusion and exclusion criteria, have the capacity to understand the written informed consent form, and be willing and able to comply with the study schedule, requirements, restrictions, and COVID-19 policies. Enrolled participants, consistent with inclusion and exclusion criteria, were non-smoking males, between 18 and 55 years of age with a body weight between 73 and 99 kg at screening. Participants were required to be in good health as determined by medical history, physical examination, vital sign measurements, 12-lead ECG, and clinical laboratory test results.

The male study participants were required to agree to follow the protocol requirements concerning use of contraception and sperm donation.

Screening serology results for hepatitis B surface antigen, hepatitis C virus antibody, and human immunodeficiency virus antibody were required to be negative. Negative urine test results for both drugs of abuse and alcohol, at screening and admission, were required. The content of ^14^C in urine and whole blood samples obtained at screening were required to not significantly exceed the general environmental background ^14^C level.

Exclusion criteria included a recent (within 60 days) large blood loss or donation of bone marrow or plasma. Receipt of any IP or investigational device within 30 days (or 5 half-lives) of dosing, whichever was longest, was also exclusionary. Also, prospective participants who participated in another clinical study with a ^14^C labelled drug administered within 1-year were not permitted to enrol. Other exclusionary criteria included recent or new use of alcohol, food containing xanthines or caffeine, excessive exercise, use of cannabis or cannabidiol, smoking, tobacco use, nicotine-containing products, grapefruit-containing foods or beverages, and Seville orange-containing foods. Use of prescription and non-prescription drugs, antacids, herbal remedies, and vitamins was proscribed from the screening encounter until study Day 11. Acetaminophen at a dose ≤1 g/day was permitted if approved by the Investigator.

### Sample collection and processing

#### Whole blood

Serial blood samples were collected to determine zalunfiban and its major metabolite des-gly-zalunfiban (M1) whole blood concentrations, additional whole blood metabolite profiling, and total whole blood ^14^C at pre dose (−60 min); 5, 10, 15, 30 and 45 min; and 1, 1.5, 2, 2.5, 3, 4, 6, 8, 12, 16, 24, 36, 48, 72, 96, 120, 144, 168, 192, 216 and 240 h after dosing. Blood samples were taken *via* an indwelling IV catheter or by direct venipuncture into designated blood collection tubes, with K_2_ EDTA used as an anticoagulant. Study samples were diluted immediately after blood collection, at a ratio of 1:4 using prechilled 30% acetonitrile, then separated into storage tubes and immediately frozen at −80°C for shipment to the analytical laboratory. At the laboratory, extraction of ^14^C-zalunfiban from whole blood was achieved by repeated microcentrifugation, vortex mixing, and harvesting to collect analytes (see Metabolite profiling and excretion for more details). Note that whole blood, rather than plasma samples, were collected as earlier human studies demonstrated that zalunfiban concentrations in whole blood were consistently higher and less variable.

#### Urine and feces

During screening, 1.0 mL aliquots of urine were collected from urinalysis samples and used for a background ^14^C measurement.

Following admission, urine was collected pre-dose, and then post-dose over the following collection time intervals: 0–4, 4–8, 8–12, 12–24, 24–48, 48–72, 72–96, 96–120, 120–144, 144–168, 168–192, 192–216, and 216–240 h. During each interval, all individual urine samples were collected, and retained samples harvested and stored at 2–8 °C. Harvested urine samples were then pooled in 3,000 mL collection containers. From each container 4.5 mL and 12 mL aliquots were obtained for ^14^C analysis and metabolite profiling and identification, respectively.

Individual bowel movements were collected in labelled containers and stored at 2–8 °C. These were pooled into containers as per the following collection intervals: pre-dose and post-dose 0–24, 24–48, 48–72, 72–96, 96–120, 120–144, 144–168, 168–192, 192–216 and 216–240 h. Samples were harvested, homogenised with deionised water at a ratio of 1:1 to 1:2, and stored at 2–8 °C (See Metabolite profiling and excretion for more details). Approximately 4.5 mL of homogenate was retained for ^14^C analysis and 12 mL retained for metabolite profiling and identification.

Whole blood samples were analysed using accelerator mass spectrometry (AMS). Urine sample analysis was performed using liquid scintillation counting analysis. Each sample was counted for at least 10 min or until the 2%2σ terminator was reached. Homogenised faeces samples were combusted in a Perkin Elmer, Model 307 sample oxidiser (Perkin Elmer, Waltham MA). The CO_2_ produced was trapped in a Carbosorb/Permafluor 2:1 v/v (Perkin Elmer, Waltham MA) to determine total carbon content and the ^14^C:^12^C ratio and then analysed by AMS.

A carbon content correction based upon pre-dose whole blood samples and urine samples was applied to all post dose samples. As the carbon content of homogenised faeces can be variable, all homogenised faeces samples were analysed by C,H,N using a Thermo Scientific FlashSmart elemental analyser (Waltham, MA). Urea standards were used to calibrate and normalise the C,H,N, and the carbon content of each sample was determined.

#### Analytical methods

Zalunfiban and M1 whole blood concentrations were measured using a validated LC-MS/MS method in compliance with the ICH-M10 guideline. The method utilised a Sciex 5500 mass spectrometer, Shimadzu LC30AD HPLC system, and a Shimadzu AD multiplate autosampler. Mobile phase A (MPA) was 10 mM ammonium acetate with 0.1% ammonium hydroxide and mobile phase B (MPB) was 10 mM ammonium acetate with acetonitrile:ammonium hydroxide. An Acquity UPLC BEH Hilic 1.7 micrometer column (Waters Corporation) was used at a temperature of 50 °C. The column flow rate was 0.4 mL/min and injection volumes were 2.0 microliters. The gradient for analytical pumps was set at 30% MAP and 70% MPB.

For whole blood LC-MS/MS the calibration ranges were 5.00–500.00 ng/mL for zalunfiban and 25.00–2500.00 ng/mL for M1. Zalunfiban and M1 sample concentrations below 5 ng/mL and 25 ng/mL, respectively, were reported as below the lower limit of quantification (<LLOQ).

Intra-run accuracy and precision was monitored using at least 6 quality control samples per run for all analytical runs. Acceptance criteria required that the mean precision and % relative error (RE) from nominal be within ±20.0% at the LLOQ and 15.0% at all other concentrations.

Intra-batch accuracy and precision was assessed using a minimum of 2 sample replicates at each quality control (QC) level, per run. For acceptance, accuracy from nominal was required to be within ±15.0% RE for at least 50% of the QC samples at each concentration and at least 2/3 of all QC samples within ±15.0% RE.

For metabolite profiling in whole blood, urine, and faeces, LC-MS/MS followed by AMS was used. The HPLC system included a 1290 Infinity Sampler, Binary Pump, and thermostat (Agilent, UK). The chromatographic method used an aqueous formic acid (MPA)/ammonium acetate:acetonitrile:methanol (MPB) gradient and a Scherzo SS-C18 HPLC column at a temperature of 40 °C with injection volumes of 50–200 microliters. The gradient was set at 90% MAP and 10% MPB. The mass spectrometer was a Q-Exactive system (Thermo Fisher Scientific, UK) and the AMS a single stage NEC SSAMS-250.

Single whole blood, faeces, and urine pools were created across subjects and radioactive regions representing 10% or more of the circulating radioactivity were analysed (e.g. 10% or more of the circulating radioactivity in whole blood or accounting for 10% or more of the administered radioactivity/dose in urine and faeces), To create the pool, an individual subject AUC pool was prepared, and a constant proportion of each subject pool was taken to create one cross-subject pool. The whole blood sample pool included samples from hours 0–144, the urine sample pool included samples from hours 0–48, and the faeces pool from hours 0–192.

Whole blood, faeces, and urine radiochromatograms were generated for each sample by collecting HPLC fractions at intervals of 2 (30 s) or 4 (15 s) per minute over the run time of 110 min.

Radiochromatograms were generated from AMS data using the LAURA V6 software (LabLogic, Sheffield, UK). Regions of interest were quantified and provisionally identified where possible by assignment of observed peaks in each radiochromatogram to either parent zalunfiban or M1 by retention time matching, with corresponding ^12^C-reference standards obtained *via* LC-UV.

Assignment of parent and metabolites was made by matching retention times with synthesised standards at wavelengths of 210, 230, 254, 270, and 300 nm, using liquid chromatography ultraviolet chromatography (LC-UV).

For urine and faeces samples, total radioactivity analysis was initially performed using two liquid scintillation counters (LSC), a TriCarb 3180 TR/SL and a TriCarb 5110 TR (Perkin Elmer, Waltham MA). When the measured total radioactivity concentration was above the LLOQ, LSC data were reported. For LSC, the LLOQ was defined as twice the background count rate. If the radioactivity concentration was below the limit of quantification (BLOQ), samples were analysed by Accelerator Mass Spectrometry (AMS). For AMS analysis, the LLOQ for total radioactivity depends on multiple factors, including the carbon content of the sample, and it is provided as the mean of all participant values. The mean (SD) total radioactivity LLOQ for zalunfiban in whole blood was 0.2421 (0.0571) ngEq/mL, for faeces 0.0663 (0.0135) ngEq/gm, and urine 0.0088 (0.0029) ngEq/g.

All samples analysed were within the established stability window for each analyte.

#### Sample processing

For extraction of ^14^C-zalunfiban from whole blood, weighed aliquots in microcentrifuge tubes were processed with vortex mixing and centrifugation. The supernatant was then harvested and the process repeated with the bullet material in the original tube using acetonitrile and then methanol. The combined aliquots then underwent AMS analysis to determine the extraction recovery. Once optimised, the final combined supernatant was dried under N_2_ gas at 40 °C and reconstituted with 1 mL methanol:water (20:80, v/v), with vortex mixing and sonication. This was followed by analysis by LC. The radioactivity extraction recovery for whole blood (AUC0–144h) was 54.5%,

For extraction of ^14^C-zalunfiban from homogenised faeces, weighed aliquots in microcentrifuge tubes were processed with vortex mixing and centrifugation, and then diluted with acetonitrile, followed by additional mixing, centrifugation, and repeat extraction with methanol:water until optimised. Once optimised, the final combined supernatant was dried under N_2_ gas at 40 °C and reconstituted with methanol, followed by water, followed by vortex mixing, followed by analysis by LC. The radioactivity extraction recovery for faeces (0–192 h) was 100%.

Urine was not subjected to extraction. An aliquot was dispensed into an LC vial prior to analysis.

The internal reference standards used were either deuterated zalunfiban or deuterated M1,

### Pharmacokinetic analysis

Zalunfiban and whole blood radioactivity concentrations were used to calculate PK parameters using standard non-compartmental analysis (NCA) methods using Phoenix WinNonlin software (Phoenix, Princeton, NJ). All statistical programming and tables, figures and listings were carried out using SAS 9.4 (SAS Institute, Inc., Cary, NC).

### In vitro testing for inhibition of platelet aggregation

Ten mL of whole blood was collected from healthy human donors *via* venipuncture and treated with an anticoagulant (D-phenylalanyl-prolyl-arginyl chloromethyl ketone). From this, platelet-rich plasma (PRP) was prepared by centrifuging whole blood at room temperature at 650 × g for 4 min. PRP was removed and standardised to a uniform platelet concentration of 3.0 × 10^5^/μL, using platelet poor plasma as a diluent, where necessary. M1 was tested for inhibition of platelet aggregation *via* incubation with PRP for 20 min at 22°C followed by the addition of 25 μL of 50 μM adenosine diphosphate to initiate aggregation. Platelet aggregation was measured using an aggregometer (PAP-8E C/N 106075; Bio/Data Corp, Horsham PA). For M1 a range of concentrations was tested to produce a single fitted curve, and the Levenberg-Marquardt approach was used to calculate IC50 values.

### Safety assessments

The study was reviewed, approved, and monitored by both an independent Institutional Review Board and a Data Safety Monitoring Board, and the protocol was performed under an Investigational New Drug Application. The safety of single-dose SC zalunfiban was evaluated by monitoring adverse events, clinical laboratory data, vital signs, 12-lead electrocardiogram, and physical examination. Vital signs (blood pressure, heart rate, body temperature, respiratory rate) were measured at screening, admission, pre-dose, post-dose at 1, 4, 8, hours, and in the morning on study days 2–11. Safety laboratory tests included serum chemistry, haematology, coagulation, and urinalysis, and were performed at screening, admission, and on study days 1, 4, and 11. On day 1, additional platelet counts were performed post-dose at 2, 6, and 12 h. Following SC administration, the injection site was observed for bruising and/or haematoma. Participants were continuously monitored for adverse events (AEs) and new use of medication(s).

During the study, AEs were collected from the time of informed consent to the last time the participant was seen. Any AE that occurred after the start of study drug administration was considered a treatment-emergent AE (TEAE). The investigator or designee assessed the severity of all AEs using the toxicity grading scale in National Cancer Institute Common Terminology Criteria for Adverse Events v5.0. These scales assign a grade of 1 through 5 to indicate the severity of AEs.

## Results

### Enrolment and demographics

Eight male volunteers were enrolled as planned without need for alternate participants. All participants received the per protocol dose of SC ^14^C-zalunfiban, and the entire dose was administered without significant loss at the injection site. All participants underwent all of the planned study protocol procedures and completed the study per protocol. As a result, all participants are included in both the safety and the PK populations.

The demographics and baseline characteristics of the participants are provided in Table 2.

### Pharmacokinetics

**Figure 1.**
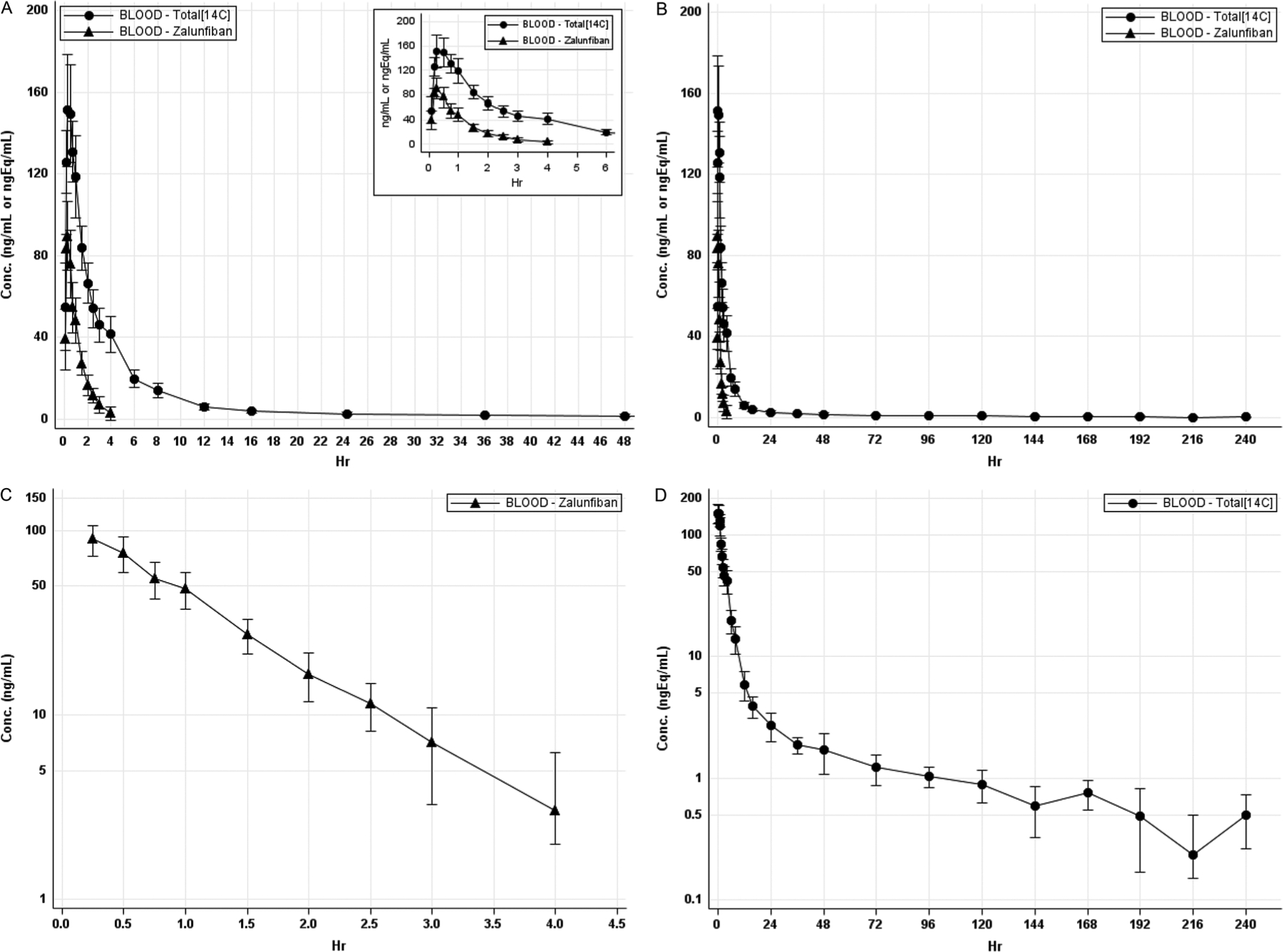
(A) Zalunfiban and total ^14^C in whole blood, concentration vs time out to 6 and 48 h. (B) Zalunfiban and total ^14^C in whole blood, concentration vs time out to the last quantifiable sample of whole blood radioactivity. (C) Zalunfiban semilog plot of concentration vs time to the last quantifiable sample. (D) Total ^14^C in whole blood concentration vs time to the last quantifiable sample.

Zalunfiban was detected in the first post-dose whole blood sample in all participants at 5 min. Four of eight participants had their last measurable whole blood zalunfiban concentrations at 4 h post-dose, 3 of 8 participants had their last measurable whole blood concentrations at 3 h post-dose, and one participant had a last measurable concentration at 2.5 h post-dose (Figure 1).

Whole blood zalunfiban concentrations reached a peak by approximately 0.25 h (median) and thereafter declined with a mean (SD) terminal T_1/2_ of 0.96 (0.33) hours. The mean (SD) C_max_ of zalunfiban was 95.23 (11.52) ng/mL, mean (SD) AUC_0-last_ was 109 (19.6) h*ng/mL and mean (SD) AUC_0-inf_ was 118 (19.5) h*ng/mL (Table 3). These results are consistent with what has been observed in earlier pharmacokinetic studies (Kereiakes et al. 2020).

As with zalunfiban, whole blood radioactivity was detected in all participants at the 5 min post-dose blood sample. Peak whole blood radioactivity occurred at 0.50 h (median) and thereafter declined in an apparent biphasic manner, with an overall (0.5 h to 240 h) mean (SD) T1/2 of 98.32 (31.35) hours (Figure 1A,C). The two phases are quite different (Figure 1D) with the first phase declining with an approximate half-life of 1.89 h (between 0.5 to 4 h) and the second phase declining much more slowly with an approximate half-life of 44.04 h (between hours 6 to 240). The ability to measure whole blood total radioactivity at concentrations far below the LLOQ for zalunfiban explains this finding. For whole blood radioactivity, the mean (SD) Cmax of whole blood total radioactivity was 165 (16.1) ngEq/mL, the mean (SD) AUC0-last was 705 (81.6) h*ngEq/mL and the mean (SD) AUC0-inf was 782 (102) h*ngEq/mL (Table 3). For whole blood total radioactivity, total radioactivity remained quantifiable up to 240 h post-dose in all subjects, except for one where the last measurable concentration was at 216 h post dose (Figure 1B,D).

Mean whole blood zalunfiban/total radioactivity expressed as a percentage was 35.6% for AUC_0–4_ (the last time at which both zalunfiban and ^14^C-zalunfiban were detectable), and 15.1% for AUC_0-inf_ (Table 3). This last value has to be interpreted with caution due to the vast difference between the sensitivity of the analytical method for zalunfiban (when it can only be detected for 4 h) and that of ^14^C-zalunfiban (where it is detectable for 240 h).

### Excretion

**Figure 2.**
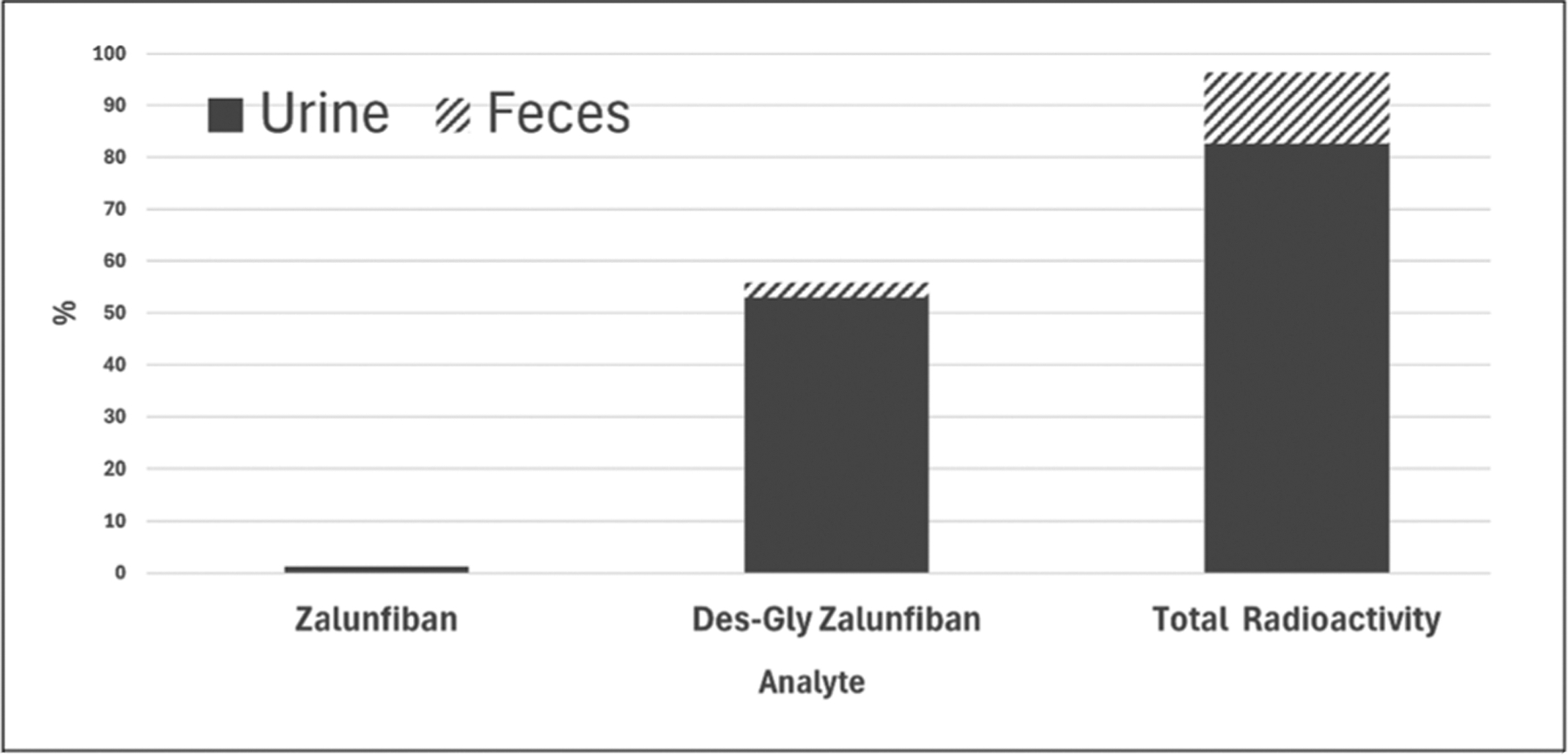
Cumulative mean dose recovery of zalunfiban, M1 and total radioactivity, by analyte and route of elimination*. *Collection times: zalunfiban and M1 in urine (0–48 hours), faeces (0–192 hours), total radioactivity (0–240 hours).

After a single SC dose of ^14^C-zalunfiban, cumulative percentages of recovered ^14^C ranged from 94.6 − 98.1% by 240 h post-dose, with cumulative faecal recovery ranging from: 10.7% − 18.5% and cumulative urine recovery ranging from 77.1% − 86.6%. Most of the excretion took place by the 144-hour post-dose timepoint. Urine was the major route of ^14^C recovery (Figure 2).

**Figure 3.**
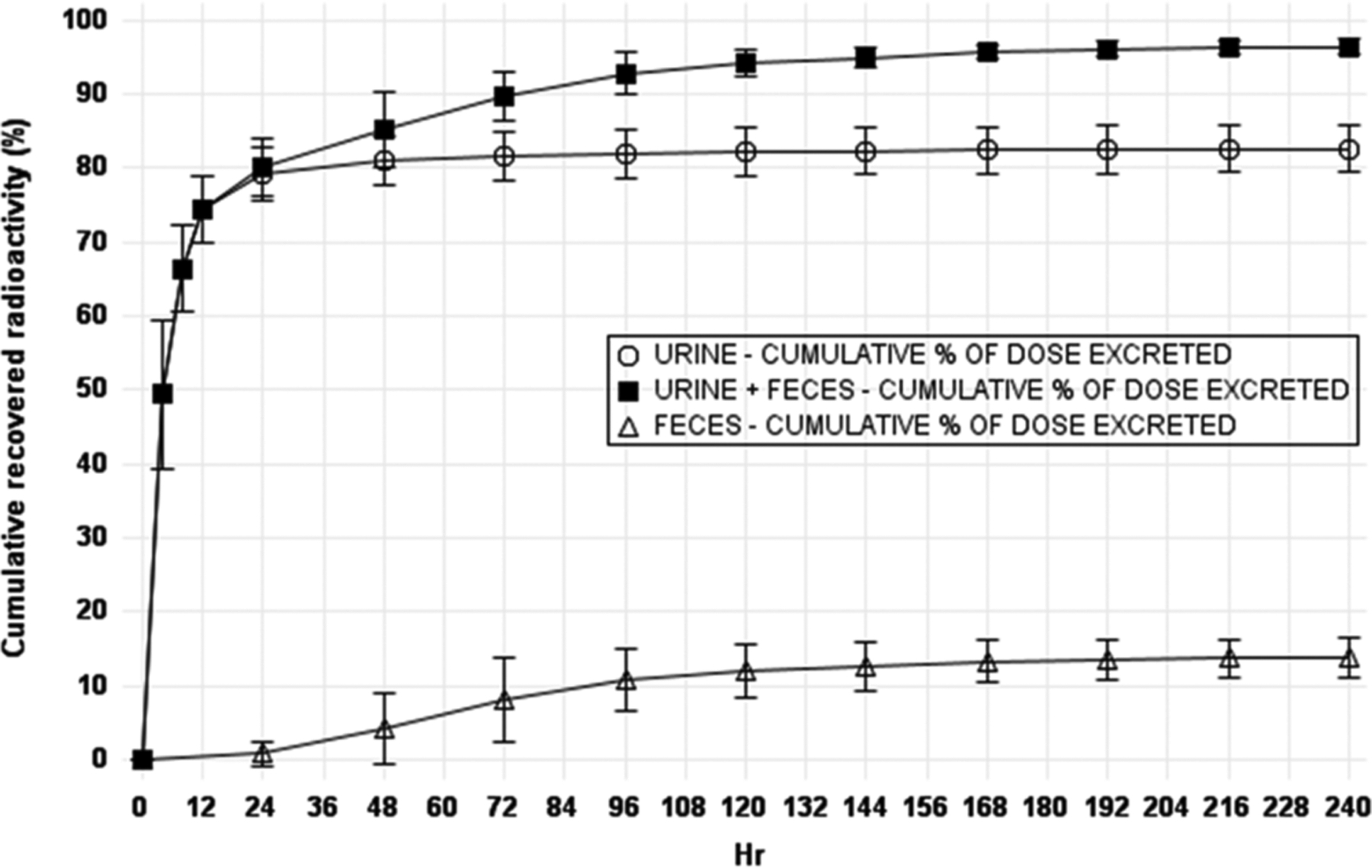
Radioactivity mean cumulative percent recovery in urine, faeces, and total, following single dose subcutaneous ^14^C-zalunfiban 5 μci/9.5 mg.

Reviewing the cumulative recovered radioactivity (%) vs time profile for urine, faeces, and total radioactivity shows that on average more than 80% of the radioactivity was recovered by 48 h, with the majority of ^14^C in urine. Excretion of smaller amounts in faeces was delayed, and this accounts for most excretion past 48 h (Figure 3).

### Metabolite profiling and excretion

In whole blood extracts, only 2 peaks with more than 5% of total radioactivity were seen in the radiochromatograms from the AUC0–144 cross-pooled sample (Figure 5A). Based on retention times, unchanged parent (zalunfiban), was identified as the major component, accounting for 46.9% of total circulating radioactivity, which is a value similar to the mean whole blood zalunfiban/total radioactivity expressed of 35.6% for AUC_0–4_. M1 accounted for 24.4% of total circulating radioactivity. Each component peak of the remaining circulating radioactivity accounted for <5% of the total.

In the faeces extracts there were 6 peaks with more than 5% of total radioactivity (Figure 5B). Based on LC-UV retention times, M1 was identified as the major radioactive component, accounting for 20.8% of the total radioactivity identified in the 0–192 h sample. This is equivalent to 2.87% of the dose excreted. Zalunfiban accounted for 0.173% of total radioactivity, equivalent to 0.0239% of the dose.

In urine extracts there were 2 peaks with more than 5% of total radioactivity (Figure 5C). Based on LC-UV retention times, M1 was the major radioactive component in urine, accounting for 64.1% of the 0–48 h cross-participant pooled sample and equivalent to 53.0% of the dose excreted. Zalunfiban accounted for 1.59% of total radioactivity, equivalent to 1.31% of dose excreted (Figure 2). Unknown component (U1), accounted for 5.60% of the total, equivalent to 4.63% of the dose excreted.

### Metabolite identification and parent drug conversion

**Figure 4.**
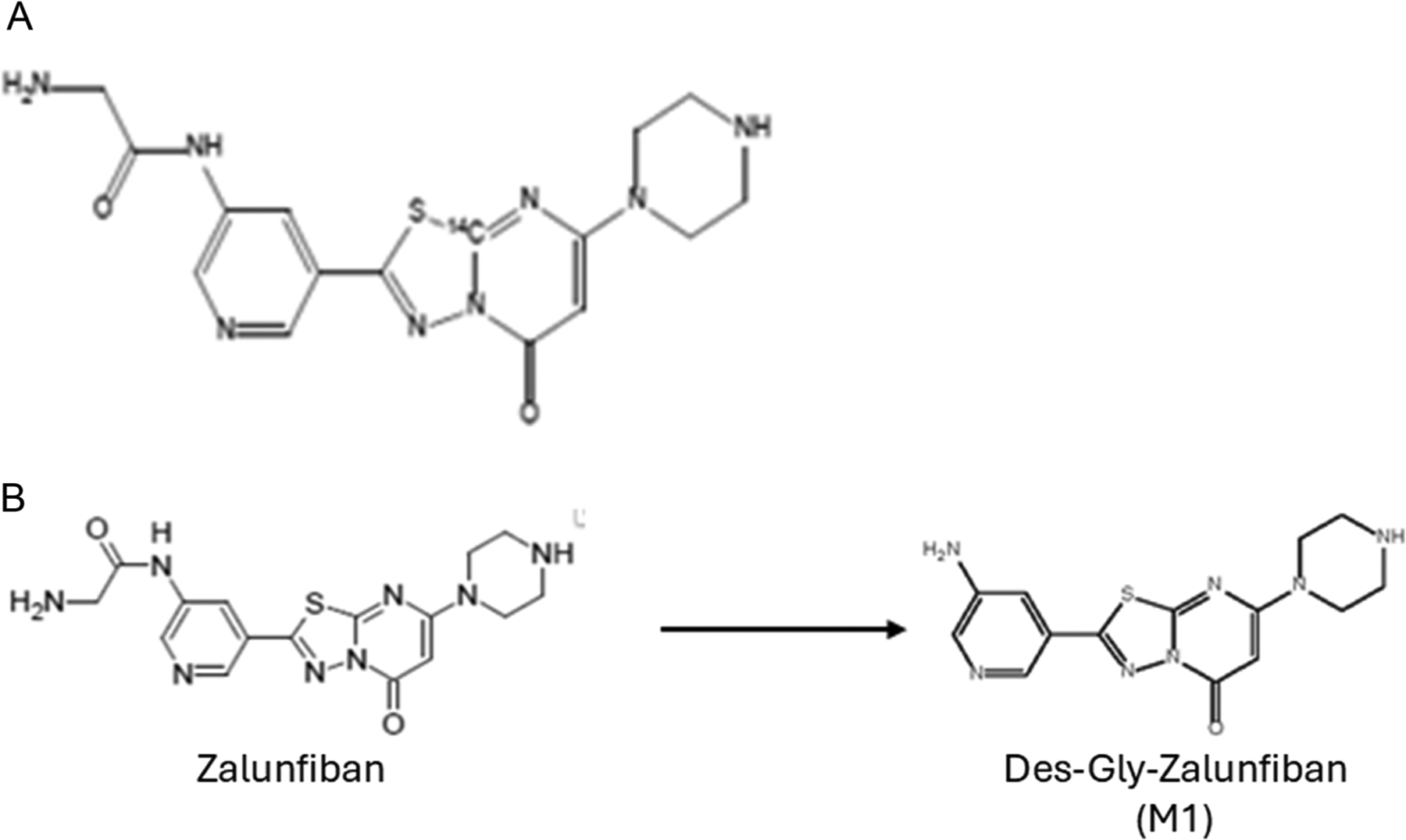
(A) Zalunfiban structure and location of ^14^C. (B) Metabolic conversion of zalunfiban to M1.

**Figure 5.**
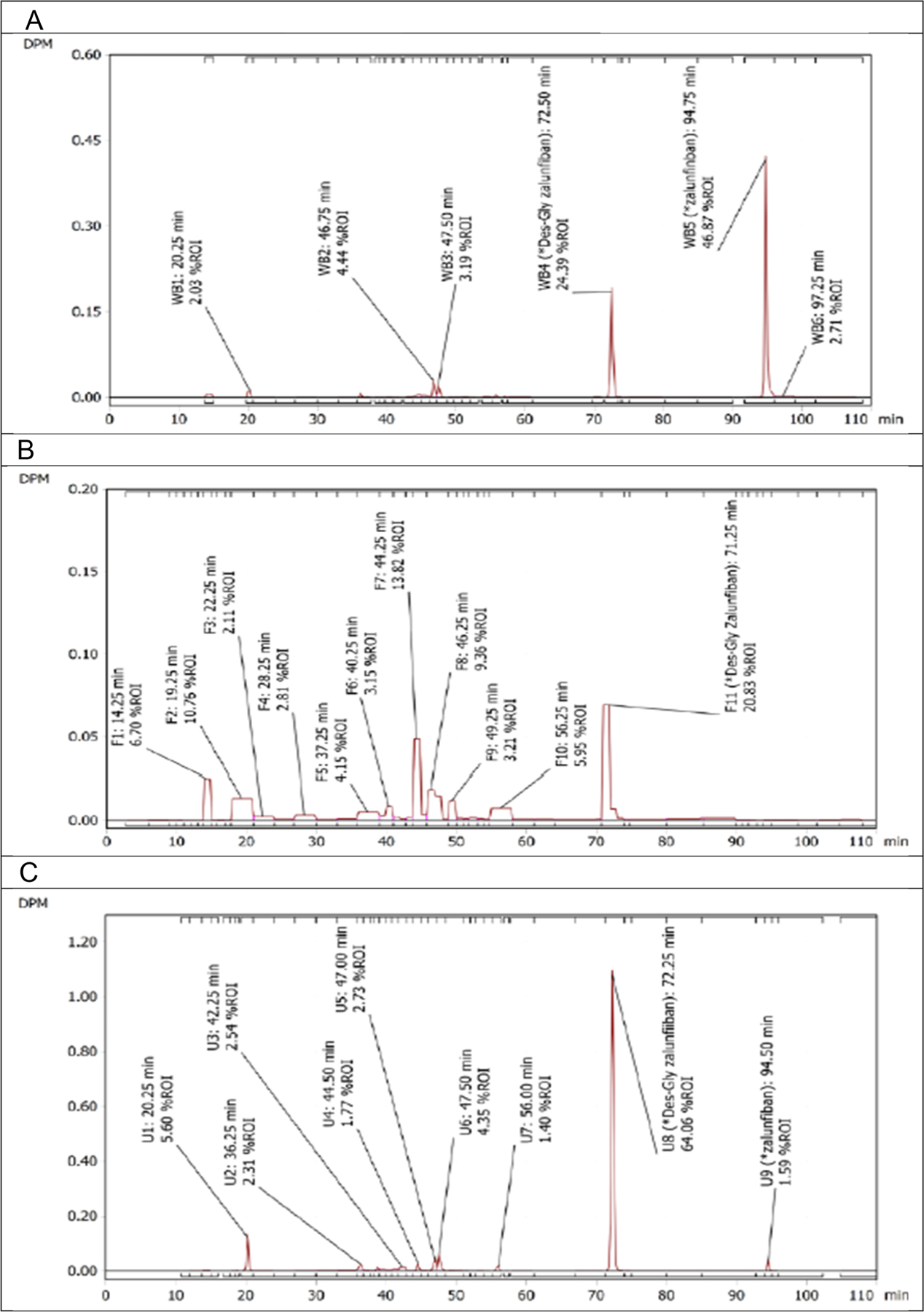
(A) Radiochromatogram of whole blood extract (AUC0–144 h). (B) Radiochromatogram of faeces extract (AUC0–192 h). (C) Radiochromatogram of urine extract (AUC0–48 h).

Plasma, urine, and faeces samples were analysed with the objective of identifying radioactive regions representing 10% or more of the circulating radioactivity in whole blood (‘AUC’ pool) or accounting for 10% or more of the administered radioactivity/dose in urine and faeces. No single radioactive region in faeces accounted for greater than 10% of the administered radioactivity/dose; therefore, no formal metabolite identification was performed. Based upon this, the proposed metabolic pathway of zalunfiban (Figure 4A) involves a single step, the removal of glycine to form M1 as depicted below (Figure 4B). This inactive metabolite is then primarily excreted in the urine, with lesser amounts excreted in the faeces.

### Antiplatelet activity of M1

The IC50 for platelet aggregation of M1 was 25.5 ± 3.5 μM, whereas the IC50 for zalunfiban was 0.112 μM, indicating that M1 has < 0.05% of the antiplatelet activity of zalunfiban. As M1 was present at very low concentrations in whole blood, all below the LLOQ of 25 ng/mL, it did not contribute to pharmacodynamic activity.

### Safety

All 8 dosed participants were included in the safety population. ^14^C-zalunfiban was safe and well-tolerated. A total of 4 participants experienced Grade 1 AEs, and 1 participant experienced a Grade 2 AE. Observed TEAEs included injection site bruising (1 participant, grade 1), injection site pain (1 participant, grade 1), headache (2 participants, 1 grade 1 and one grade 2), and confusion (1 participant, grade 1). There were no observed clinically significant changes in clinical laboratory tests, vital signs, or ECGs. There were no serious TEAEs, and no TEAEs leading to study discontinuation.

## Discussion

In this phase 1, open-label, single-dose trial, following SC injection of ^14^C-zalunfiban, radioactivity was rapidly detected in whole blood, and over 80% of the total dose was recovered in urine and faeces by 48 h. Zalunfiban was the predominant radiolabeled component in whole blood, with only one metabolite (M1) exceeding the threshold of 10% of total radioactivity whole blood AUC_0–144_, in cross-pooled samples. The primary elimination pathway for zalunfiban is the metabolic conversion to M1, which is then excreted mainly in urine and in limited amounts in faeces. Zalunfiban was not detected in whole blood after at 3.5 h (median) and it comprised only 1.31% of the dose excreted in the urine over 48 h, and 0.0239% of the dose detected in the faeces (0–192 h). Since zalunfiban is not substantially eliminated by the kidney as defined by FDA (FDA 2024b), dose adjustment in patients with reduced renal function may not be required (Tabe 1). Zalunfiban is intended for single dose administration, and FDA has stated that hepatic impairment studies generally are not useful for single dose products. (FDA 2003). That recommendation, together with the limited amount of zalunfiban detected in faeces, suggests that zalunfiban dose adjustment in patients with reduced hepatic function may also not be required (Tabe 1). Zalunfiban was well-tolerated, with no serious TEAEs and no TEAEs leading to study discontinuation. All dosed participants were included in both the PK and safety populations. One limitation of our study is that all participants were male, which is common in mass balance studies (Ramamoorthy et al. 2022).

These study results highlight important differences between zalunfiban and the GPIIb/IIIa inhibitors tirofiban and eptifibatide, which are currently available in the US. Both tirofiban and eptifibatide require dose reduction when administered to patients with moderate to severe renal insufficiency, corresponding to creatinine clearance thresholds below ≤60 mL/min and < 50 mL/min, respectively (AGGRASTAT^®^ (tirofiban hydrochloride) injection 2020; Integrilin (Eptifibatide) Injection 2013). It is estimated that 30% of STEMI patients have chronic kidney disease, as defined by an estimated glomerular filtration rate of < 60 mL/min per 1.73 m^2^ (Marenzi et al. 2012). Tirofiban and eptifibatide also have more complicated IV administration requirements, requiring administration of one or more bolus doses, followed by a continuous infusion. In comparison, zalunfiban can be administered *via* a single small volume SC injection, and so is designed for pre-hospital administration by emergency medical service (EMS) personnel or self-administration.

All key regulatory PK and drug metabolism questions previously described (Table 1) were addressed. Zalunfiban is rapidly absorbed following SC injection. The C_max_ (geometric mean) is 94.62 ng/mL (range 80.78–110.59 ng/mL), which is reached in a median time of 15 min (range 10–30 min). In parallel, the primary metabolite, M1, and other minor metabolites are rapidly formed, following a slight lag time (based upon total whole blood radioactivity), reaching a C_max_ (geometric mean), of 164 ng/mL (range 141–185 ngEq/mL) with a median T_max_ of 30 min (range 15–60 min). Mean zalunfiban concentrations were below mean total whole blood radioactivity at all timepoints where zalunfiban was measurable, and the proportion of total radioactivity due to zalunfiban is 35% over the first 4 h. While zalunfiban was detectable the mean whole blood half-life of zalunfiban is 0.96 h (range 0.64–1.66 h), which exhibited a mono-exponential decline and was no longer detected at a median time of 3.5 h. In whole blood, zalunfiban was the major analyte, based upon radioactivity values over 144 h (based upon AUC0–144), accounting for 46.9% of total circulating radioactivity. M1 accounted for 24.4% of total circulating radioactivity based upon radiochromatograms from the AUC_0–144_ cross-pooled sample, based upon LC-UV retention times. The major M1 metabolite was undetectable in blood, where the limit of quantification was 25 ng/mL. M1 has very limited water solubility (< 10 ng/mL) and is more lipophilic than zalunfiban. This may account for the much longer half-life of whole blood radioactivity and its prolonged excretion phase, when compared with zalunfiban. The whole blood AUC pool up to 144 h post-dose was considered sufficient to characterise metabolites because most excretion took place by the 144-hour post-dose timepoint.

Total whole blood radioactivity declined in a biphasic manner, with an approximate first phase half-life of 1.89 h and the second phase half-life of 44.04 h, and an overall mean half-life of 98.3 h (range 58.55–153.94 h). Note the total whole blood radioactivity assay had a much lower limit of detection when compared with the zalunfiban LC-MS/MS assay. Despite that difference, zalunfiban was still the primary whole blood analyte.

The goal of recovering over 80% of radioactivity in urine, faeces, and total combined was achieved by 48 h in this study although the sample collections continued to 240 h. Total cumulative percentages of recovered ^14^C ranged from 94.6–98.1% of the dose by 240 h post-dose, with 10.7% − 18.5% in faeces and 77.1% − 86.6% in urine. Overall, most excretion took place by the 144-hour post-dose timepoint. Urine was the major route of ^14^C recovery (Figures 2 and 3).

While zalunfiban is the primary analyte in whole blood, the prevalent analyte in urine and faeces, based upon radioactivity, is M1. Zalunfiban was detected in only small amounts in urine and faeces at 1.31% and 0.0239% of dose, respectively. Conversely, M1 accounted for 53.0% and 2.87% of the dose excreted in urine and faeces, respectively. Given the minimal amounts of zalunfiban excreted in urine and faeces, zalunfiban does not appear to meet the FDA threshold for drugs that are substantially eliminated by either the kidney or liver, suggesting dose adjustment based upon organ function may not be needed.

M1 is considered an inactive metabolite because it has <1% of the antiplatelet activity of zalunfiban. Thus, both primary metabolic conversion and deactivation of zalunfiban occurs in a single step, with the removal of glycine to form the M1 metabolite (Figure 4).

## Conclusion

Zalunfiban is a novel SC administered GPIIb/IIIa inhibitor, intended for first point-of-contact treatment of STEMI. It is primarily converted to a single inactive metabolite, M1. Elimination of zalunfiban occurs rapidly with a half-life of approximately 1 h. M1 is primarily eliminated in urine, with lesser amounts in faeces.

## Figures and Tables

**Table 1.** Key pharmacokinetic, metabolism, and regulatory considerations of a mass balance study^.

Generate whole blood concentration versus time profiles of total radioactivity, parent drug, and metabolites of interest, and identify pharmacokinetic analytes of interest.
Obtain pharmacokinetic parameters of total radioactivity, parent drug, and metabolites of interest.
Obtain cumulative percentage of administered dose radioactivity recovered in urine, faeces, and combined over time.
Identify primary metabolic and elimination pathways, where more than 80% of radioactivity is recovered in excreta.
Identify metabolite(s), including structural characterisation of metabolites representing more than 10% of total drug exposure in plasma/whole blood.
Construct a proposed biotransformation scheme based upon the structurally identified metabolites and routes of elimination.
Determine the percentage of systemically available parent drug and active metabolite(s) eliminated by the kidney. If this is 30% or more, this indicates the drug is substantially eliminated by the kidney.
Determine the percentage of parent drug and active metabolite(s) eliminated by the liver. If this is 20% or more of the absorbed drug, this indicates drug is substantially eliminated by the liver. For drugs intended for single-dose administration, a hepatic impairment study will generally not be useful, unless clinical concerns suggest otherwise.

^ FDa Guidance Mass balance Studies 2024.

^ FDa Guidance for Industry Pharmacokinetics in Patients with Impaired Renal Function 2024.

^ FDa Guidance for Industry Pharmacokinetics in Patients with Impaired Hepatic Function 2003.

^ Ducharme et al. 2022.

**Table 2.** Demographics and baseline characteristics.

Characteristic		Value
Total number enrolled		8
Sex	Male (%)	8 (100)
Ethnicity	Hispanic or Latino (%)	1 (12.5)
	Non-Hispanic or Latino (%)	7 (87.5)
Race	White (%)	2 (25)
	Black or African American (%)	6 (75)
Age	Median (range), years	37.0 (24–54)
Weight	Median (range), kg	82.50 (74.6–92.3)
Height	Median (range), cm	175.25 (167.9–188.8)
Body Mass Index	Median (range), kg/m^2^	26.20 (24.5–29.6)
Dose Administered	Median (range), mg/kg	0.115 (0.103–0.127)

**Table 3.** Pharmacokinetic parameters in whole blood in the pharmacokinetic population.

	Mean	SD	%CV	Min	Median	Max	Geom Mean	Geom %CV
Whole Blood Zalunfiban (ng/mL)								
C_max_ (ng/mL)	95.23	11.52	12.1	80.78	96.87	110.59	94.62	12.3
T_max_ (h)	0.26	0.1	39.4	0.17	0.25	0.5	0.25	34.3
t1/2 (hr)	0.96	0.33	34.6	0.64	0.84	1.66	0.92	31.8
AUC_0-last_ (hr*ng/mL)	109	19.6	18	79.9	114	141	107	18.7
AUC_0-inf_ (hr*ng/mL)	118	19.5	16.6	86.6	123	150	116	17.2
CL/F (L/hr)	81.8	14.4	17.6	62.4	76.3	108	80.7	17.2
Vz/F (L)	114	50.0	43.9	78.0	96.6	230	107	36.6
Kel (1/hr)	0.785	0.218	27.7	0.416	0.826	1.08	0.755	31.8
tlast (hr)	3.4	0.58	0.17	2.5	3.5	4	3.4	
Whole Blood Total Radioactivity (ngEq/mL)								
C_max_ (ngEq/mL)	165	16.1	9.7	141	170	185	164	10
T_max_ (h)	0.50	0.27	53.5	0.25	0.50	1.00	0.44	56.9
t1/2 (hr)	98.32	31.35	31.9	58.55	96.6	153.94	93.96	33.4
AUC_0-last_ (hr*ngEq/mL)	705	81.6	11.6	568	719	820	700	13.8
AUC_0-inf_ (hr*ngEq/mL)	782	102	13.1	605	816	897	776	13.8
Kel (1/hr)	0.0077	0.0025	32.8	0.0045	0.0072	0.0118	0.0074	33.3
Whole Blood Zalunfiban/Total Radioactivity								
AUC0–4 (ng/ngEq)	0.356	0.673	1.88	0.314	0.356	0.418		
AUC_0-inf_ (hr*ngEq/mL)	0.151	0.0247	16.3	0.121	0.151	0.203		

## Data Availability

The data that support the findings of this study are available from the corresponding author, [RM], upon reasonable request.
